# The Consumer Contextual Decision-Making Model

**DOI:** 10.3389/fpsyg.2020.570430

**Published:** 2020-09-29

**Authors:** Jyrki Suomala

**Affiliations:** NeuroLab, Laurea Leppävaara, Laurea University of Applied Sciences, Espoo, Finland

**Keywords:** inductive inference, Occam’s razor, Bayesian reasoning, consumer, decision-making, neuroeconomics

## Abstract

Consumers can have difficulty expressing their buying intentions on an explicit level. The most common explanation for this intention-action gap is that consumers have many cognitive biases that interfere with rational decision-making. The current resource-rational approach to understanding human cognition, however, suggests that brain environment interactions lead consumers to minimize the expenditure of cognitive energy according to the principle of Occam’s Razor. This means that the consumer seeks as simple of a solution as possible for a problem requiring decision-making. In addition, this resource-rational approach to decision-making emphasizes the role of inductive inference and Bayesian reasoning. Together, the principle of Occam’s Razor, inductive inference, and Bayesian reasoning illuminate the dynamic human-environment relationship. This paper analyzes these concepts from a contextual perspective and introduces the Consumer Contextual Decision-Making Model (CCDMM). Based on the CCDMM, two hypothetical strategies of consumer decision-making will be presented. First, the SIMilarity-Strategy (SIMS) is one in which most of a consumer’s decisions in a real-life context are based on prior beliefs about the role of a commodities specific to real-life situation being encountered. Because beliefs are based on previous experiences, consumers are already aware of the most likely consequences of their actions. At the same time, they do not waste time on developing contingencies for what, based on previous experience, is unlikely to happen. Second, the What-is-Out-there-in-the-World-Strategy (WOWS) is one in which prior beliefs do not work in a real-life situation, requiring consumers to update their beliefs. The principle argument being made is that most experimental consumer research describes decision-making based on the WOWS, when participants cannot apply their previous knowledge and situation-based strategy to problems. The article analyzes sensory and cognitive biases described by behavioral economists from a CCDMM perspective, followed by a description and explanation of the typical intention-action gap based on the model. Prior to a section dedicated to discussion, the neuroeconomic approach will be described along with the valuation network of the brain, which has evolved to solve problems that the human has previously encountered in an information-rich environment. The principles of brain function will also be compared to CCDMM. Finally, different approaches and the future direction of consumer research from a contextual point of view will be presented.

## Introduction

The sheer number of consumption opportunities on the market outweighs consumers’ ability to assess them. This limitation to human mental capacity is a problem for most decision-making models. First, traditional models for consumer decision-making (Samuelson, 1938; Luce and Raiffa, 1989; Barry and Howard, 1990) assume that people are driven by explicit reasoning across all options. These models simply conceptualize consumer decisions as a matter of choosing the best option from those available (Kőszegi, 2010). Furthermore, these models assume that people respond only to the features of the options available to them independent of context and unaffected by other available alternatives or temporal order. These models also assume that consumers’ preferences are invariant and that they follow principles of transitivity and other axioms presented on rational choice theory (Samuelson, 1938; Luce and Raiffa, 1989). Despite the use of sophisticated axiomatic and formal framework (Luce and Raiffa, 1989), these traditional models have limited capacities to decode the intentions and thoughts driving consumer behavior in the real market. The observed behavior of consumers is much more complex than these traditional models assume (Dijksterhuis et al., 2006).

Second, behavioral economic models have shown that consumers often violate the basic axioms of traditional models (Kahneman and Tversky, 1979; Tversky and Kahneman, 1991). According to behavioral economic models, human decision-making behavior is systematically biased (Tversky and Kahneman, 1991; Shafir and LeBoeuf, 2002; Bottom et al., 2004) and “predictably irrational” (Ariely, 2009). Behavioral economics therefore has a low opinion of human rationality (Palokangas and Suomala, 2017).

Both the traditional and behavioral economic models assume that the aim of consumer decision-making is the recovery of real-world options – that is, objective consideration of sets of commodities. They assume that a consumer enters the market environment as a *tabula rasa,* and that the representation process only begins after an objective marketing stimulus or sets of marketing stimuli are presented. Then the task of the consumer’s mental system is to generate a representation of the exact properties and attributes of commodities in the market environment. Personal goals, previous consumption history, and contextual factors have been regarded either as irrelevant (by traditional models) or as sources of cognitive bias (by behavioral economic models).

These models have several problems. First, they are insufficient to identify consumers’ prior beliefs about different market contexts. This is particularly evident in the fact that most new products fail in the market. Second, they suffer from a lack of ecological validity because their basic arguments are based solely on mathematical axioms (Luce and Raiffa, 1989) and strict experimental settings (Kahneman and Tversky, 1979). The argument is not that mathematical axioms and experimental settings are the problems *per se*. Advocates of traditional models have proofed consumer decision-making in sophisticated ways, but the explanation power of these models is frustratingly limited. The problem is more that these models explain consumer decision-making in a way that is not really what decision-making in the real market looks like. Therefore, the goal of this article is to build a more plausible framework for the description and explanation of consumer behavior in real market contexts.

**Figure 1:**
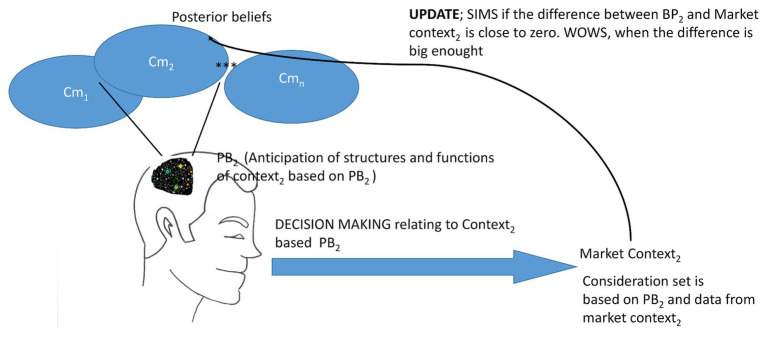
Consumer’s Contextual Decision-Making Model (CCDMM). A consumer makes decisions in the market based on his mental models of different contexts. Cm_1_, Cm_2_ …. Cm_n_ mean a contextual model_1_, a contextual model_2_, and a contextual model_n._ Let us assume that the consumer remembers that his daughter’s birthday is coming soon. This memory activates the contextual model for “my daughter’s birthday party,” and this is described as Cm_2_, which again activates prior beliefs PB_2_ and the mental construct of anticipations about a child’s birthday party. The PB_2_ activates the consumer to run an errand, in this case a visit to a department store. There, the consumer purchases gift/gifts and other supplies for the daughter’s birthday. If the PB_2_ corresponds to the anticipation of commodities (consideration set relating to the daughter’s birthday party), the update process leads to the posterior belief, which is the same as prior belief. In this case, the consumer applies SIMilarity Strategy (SIMS). However, if something in the department store is better or more interesting than PB_2_ based expectations, posterior belief will be updated, and will be different from prior beliefs. For example, if some new ice cream product for a children’s birthday party is available, the person might begin to think that it is good idea to also provide ice cream at the birthday party. When there is big enough difference between expectations and data in the market, the consumer uses the What-is-Out-there-in-the-World Strategy (WOWS), and updates his prior beliefs relating to the daughter’s birthday party. Of course, updating can go also in the negative direction.

According to more recent contextual models of human decision-making, the brain infers based on prior experience and expectation, not only of observable goods but also of the latent causes of these goods and whole context (Baum, 2004; Gershman and Niv, 2013; Büchel et al., 2014). When real market contexts include more information than the consumer can process, the brain needs to apply an effective strategy to concentrate only on meaningful information. In order to build a more plausible model of consumer decision-making, recent work has sought to identify shared principles in the mechanisms underlying subjective valuation and sensory perception (Louie and Glimcher, 2012; Woodford, 2012; Polanía et al., 2019). These contextual models have suggested that mental representation resembles sensory perception in that they are both made up of inference processes that exploit information on the relevant properties and occurrences of commodities in the environment (Polanía et al., 2019). In short, the role of the mental system of a consumer in a decision-making situation is not to represent the physical world, but to promote useful behaviors. It is essential to note, however, that a context is simply a platform on which the valuation of commodities and decision-making pertinent to coping with life are tested (see Purves et al., 2015). Decision-making reflects subjective meaningfulness based on experience rather than objective features of the environment. The goal of this article is to clarify this idea on a conceptual level. To this end, the Consumer’s Contextual Decision-Making Model (CCDMM) is presented on a conceptual level in section The Consumer’s Contextual Decision-Making Model (Figure 1). The model was constructed based on contextual and resource-rational models of human behavior and decision-making (Griffiths et al., 2015; Tymula and Plassmann, 2016; McKenzie et al., 2018).

The remainder of this paper is organized as follows: section Consumers Control Contexts by Expectations seeks to explain consumer decision-making from a contextual perspective. Before presenting the CCDMM itself, three basic theoretical constructs will be presented. First, inductive reasoning is humans’ unique capacity for extracting meaningful mental representations from sparse data. Second, the principle of Occam’s Razor will be introduced. This principle states that people prefer a simple explanation of the world over a complex one. The third basic principle behind the CCDMM is Bayesian reasoning. According to this principle, people have an existing internal model of the environment based on actions previously carried out in that environment. These existing beliefs help consumers to anticipate and interpret the structure and functions of the market. Subsequently, typical sensory illusions are presented as an example of the human capacity to mentally adapt to different contexts. Finally, the CCDMM itself (Figure 1) is presented. Section SIMS, WOWS, and Contextual Rationality begins with an exploration of two main decision-making strategies, the SIMilarity-Strategy (SIMS) and the What-is-Out-there–in-the-World-Strategy (WOWS). Both are justified by the CCDMM. A new interpretation of the concept of human rationality is then introduced and justified. Section CCDMM and Neuroeconomics discusses the most common intention-action gaps based on this new interpretation. In the same vein, framing and decoy effects will also be presented. Following this, the most typical neuroeconomics research is reviewed from the CCDMM perspective, and why the brain’s valuation network produces rational and adaptive signals to help individuals to cope in a wide variety of contexts is also explained based on the CCDMM. The article culminates in a brief discussion.

## Consumers Control Contexts by Expectations

The world around a consumer is complex and noisy, and it includes many uncertainties. Imagine an everyday situation in which a consumer goes to a department store. A typical department store has over 100,000 products. Whereas offers in this real market context represent more information than the consumer can access, the brain needs to apply an effective strategy to concentrate on the most essential information. This strategy is illustrated by the CCDMM, for which inductive inference, the principle of Occam’s Razor, and Bayesian inference are the three building blocks.

### The Three Building Blocks of Consumer Decision-Making

#### Inductive Inference

One of the greatest enigmas of human behavior is how experience leads to the formation of general and abstract knowledge. Inductive inference refers to people’s ability to infer a general principle based on observation of particular instances. Inductive inferences go beyond available data in order to arrive at plausible conclusions given what is available (Noonan, 2007; Quine, 2013). Humans are predisposed to divide the world up into objects, to understand the interactions that occur between these objects, and to apply a variety of attitudes and values to the representation of these objects. Inductive inference helps people to identify the most meaningful things about their environments (Baum, 2004). If consumers are indifferent about which goods are better than others, they will have no way of choosing the “better” good, nor will they have any way to learn (Baum, 2004).

While the origins of inductive inference are still a matter of debate, it is clear that this ability is emerges early during a person’s development and plays an important role in learning, thought, and decision-making. Thus, inductive inference is essential to the human capacity for deriving general knowledge about the structure and function of different environments from sparse data. Consider that just a few examples are enough for children to learn the meaning of certain words (Gärdenfors, 2014), causal reasoning (Gopnik and Sobel, 2000), property induction (Madole and Cohen, 1995), and social cognition (Tomasello, 1995; Gärdenfors, 2014; Lake et al., 2017).

One of the most important features of inductive reasoning is that people can learn even faster if they combine their own experience with just a little help from others (Lake et al., 2017). In social contexts, people do not only learn solely based on observations of what other people do, but also of what they do not do. As such, consumer behavior can depend on unchosen latent alternatives (Gershman et al., 2010; Kőszegi, 2010). This negative evidence and latent reasoning teach people how to avoid – without direct experience – indifferent or negative aspects of different contexts. This kind of latent reasoning as part of inductive inference without direct personal experience is almost completely missing from traditional and behavioral economic models alike. This review of inductive reasoning leads to the conclusion that the concept of meaningfulness is the strongest trigger of consumer choice, though the content of meaningfulness is different among individuals from different cultural contexts.

#### The Principle of Occam’s Razor

The fourteenth-century English theologian William of Occam’s famous advice to scientists was “not to multiply entities beyond necessity” (William and Brown, 1990). This principle, that an explanation of facts should be no more complicated than necessary, is widely accepted in the fields of science (Jaynes, 2003) and algorithmic information theory (Li and Vitányi, 2008). Though the principle is not commonly used to describe and explain human behavior, it has been recently applied to cognitive science (Gershman and Niv, 2013; Griffiths et al., 2015) and studies in preference-based decisions (Polanía et al., 2019). In addition, studies of sensory systems (Heeger et al., 1996; Barlow, 2012) and the brain valuation network (Tymula and Glimcher, 2016; Steverson et al., 2019) epitomize the original concept of Occam’s Razor.

Furthermore, the principle is central to human thought processes themselves. By analogy, just as science seeks a simple explanation for as many observations as possible, so do humans in their thought processes; people are like scientists seeking a simple explanation of the world, which, once found, is embodied in the mind (Baum, 2004). Baum (2004) takes this idea even further and argues that evolution has identified and saved simple rules into humans’ DNA, and that these rules work well in many contexts. From this perspective, our understanding of the world is a very compressed representation of it.

In addition, Occam’s principle has been used to explain category learning (Sanborn et al., 2010; Gershman and Niv, 2013). Evidence from category learning suggests that humans assign stimuli to a small set of categories, only inventing new ones when stimulus statistics change radically. For example, Gershman and Niv (2013) had participants estimate the number of colored circles on a computer screen, with the number of circles drawn from a color-specific distribution. When the color-specific distributions overlapped substantially, participants’ estimates were biased toward values intermediate between the two means, indicating that subjects grouped different-colored stimuli into one perceptual category. The study showed that humans favor simpler explanations of sensory inputs.

Consumers exhibit clear behavior when they decide which goods or services are suitable to their needs and interests. The principle of Occam’s Razor can therefore be extended to consumer behavior, as we may hypothesize that the simplest choice to accomplish a consumer’s subjective goal is the best one. Occam’s principle also has deep connections with Bayesian reasoning (Baum, 2004), which is the focus of the following section.

#### Bayesian Reasoning

The idea, that uncertain state of the world can be modeled by a prior belief with observed data, is created by Protestant theologian and minister Thomas Bayes. He lived on the eighteenth-century in England and is today such a famous name for in mathematics and statistics. This article explains consumer decision-making by applying Bayesian approach on conceptual level. Human behavior relies heavily on anticipating future states according to meaningfulness in an uncertain environment and on maintaining appropriate actions to achieve personally meaningful goals. In a real market context, the degree of uncertainty about possible outcomes will escalate drastically as the number of products and services available to a consumer increases. If examined from the perspective of the Bayesian approach, however, the consumer has recourse to an existing internal model of the environment, which can be used to anticipate and interpret its structure and function. This internal model includes both innate and adaptive components. However, it is difficult to separate these components entirely, because the innate components are also highly adaptive and the adaptive component includes mechanisms that are innate (Wilson et al., 2018). When encountering a new context, then, the consumer may infer the degree to which the information in a situation corresponds to his or her anticipation. According to Bayesian terminology, a consumer “counts” the likelihood (i.e., the probability) of the data given a hypothesis. If the information (data) of the situation does not correspond to the consumer’s anticipation (hypothesis), mental disequilibrium arises and the individual must update his or her internal model. Subsequently, this update leads to a posterior model of the context (Jaynes, 2003; Baum, 2004; Kording, 2014). Within Bayesian statistics, a previously acquired mental model is called the *prior*, while the discrepancy between new information and the prior is called the *likelihood*. According to the Bayesian approach, humans have an internal model of given contexts, which helps them to navigate different situations.

To reiterate, according to the Bayesian approach, people use prior knowledge to calculate the probability of a related event. The Bayesian approach has become increasingly important both as a tool in many areas of science (Jaynes, 2003; Lake et al., 2017) as well as a model for human learning and behavior (Friston et al., 2006; Kemp and Tenenbaum, 2008; Dasgupta et al., 2018). However, its application to consumer decision-making is less common. The Bayesian approach is a realistic and dynamic tool for understanding human behavior and learning mechanisms in the context of consumer behavior. When consumers encounter new or unexpected information, they are required to actively reformat it based on prior beliefs and new data to better serve their personal goals. When consumers receive even more data, posterior beliefs become new priors. This cycle continues indefinitely as people are continuously updating their beliefs, which is valuable to understanding decision-making. The Bayesian approach assumes that the mind inverts the internal model to compute expectations about the state of the environment. It is important to emphasize that the state of the environment includes objects, their interactions, and their latent and perceptual causes (Gershman et al., 2010; DuBrow et al., 2017).

The Bayesian approach includes one important aspect about reasoning during decision-making. The accuracy of human judgment does not only depend on how accurately an individual manipulates information in an environment, but also on how well the information corresponds to prior experience and knowledge. This prior experience gives rise to a form of expectation based on prior belief, in which people are more accurate when asked to make decisions about “believable” problems than about “unbelievable” problems, even when the logical form of these problems is equal. This is an important point, because traditional and behavioral economic models are almost silent about the prior beliefs of a consumer, and assume that the consumer logically reasons, in the classical deductive inference sense, in a market context. However, a consumer tries to make sense of the context based on prior beliefs and expectations about meaningfulness.

Despite the fact that many studies have found that people’s judgments and decisions are very closely aligned with Bayes’ optimal prediction (Griffiths and Tenenbaum, 2006), people also violate these norms (Dasgupta et al., 2018). The reason for this violation might be the curse of dimensionality: the more information and dimensions an environment has, the more combinations of features or states consumers have to learn. This makes consumers decision-making prohibitively “expensive” in terms of the amount of experience needed to master all information in a specific context.

Economics and the free market economy have long assumed that the more options there are, the better. However, this traditional view runs counter to empirical evidence that consumers make worse decisions and are more disappointed with their decision when the number of choice sets increase (Iyengar and Lepper, 2000; Iyengar and Kamenica, 2010).

Thus, consumers make approximations about the most essential features of the environment and then compare the data to their mental model according to the Bayesian principle. At least in part, the Bayesian approach solves the curse of dimensionality (Dasgupta et al., 2020) if it is assumed that inductive inference addresses and learns the most important aspects of the individuals’ environment. In this sense, cultural habits, social norms, and attitudes play an important role in consumer decision-making.

Inductive inference, Occam’s Razor, and Bayesian learning illuminate the dynamics of the human-environment relationship and are the three building blocks of the CCDMM. Respectively, they indicate that the concept of meaningfulness is the strongest trigger of consumer choice, that the best means of accomplishing this choice or goal is the simplest one, and that cultural habits, social norms, and attitudes play an important role in consumer decision-making.

### Sensory Perception as an Illustration of the Sophisticated Human Mental System

The study of how properties of context is represented in the brain mainly begins with the study of sensory systems. These studies have shown that the activity of neurons in the sensory areas of the brain is conditional on the expectation of future properties of stimuli cortices (Tymula and Glimcher, 2016; Heeger, 2017). Recently, neuroeconomics studies have found that the human brain – especially its valuation network – is dynamically adjusted in response to expectations (Woodford, 2012). The physiological responses of sensory neurons show outcome expectations that match the manner in which models of decision-making predict expectations according to utility functions (Woodford, 2012; Tymula and Glimcher, 2016). Therefore, extensive evidence of these studies of sensory perception have identified the fundamental primitives of many common neurophysiological functions and representations, which are sensitive for predictions not only in the sensory areas, but also in the valuation areas in the brain, which drives the human decision-making process (Tymula and Glimcher, 2016).

At the heart of the CCDMM lies the idea that we do not interpret our world merely by analyzing incoming sensory information, but rather we try to understand it by proactively linking incoming sensory information to familiar prior beliefs (Bar, 2011). In this way, our perception of the environment relies on prior beliefs as much as it does on incoming information, which blurs the border between sensory information and decision-making (Bar, 2011). The three examples of perceptual illusions describe how our brains make approximations in order to behave in suitable ways in different contexts.

Perceptual illusions are well-known phenomena in psychology (Glimcher, 2011), and scientists have applied the findings of perceptual illusions, when they have built conceptual foundations for context-dependent decision-making models (Glimcher, 2014). Whereas behavioral economists view these illusions as an indication of the human tendency toward irrational behavior (Thaler and Sunstein, 2009), recent contextual approaches have emphasized, rather, that these illusions expose the effectiveness of the human mental system (Glimcher, 2011; Tymula and Plassmann, 2016; DuBrow et al., 2017; McKenzie et al., 2018). The core lesson from studies of perception is that sensory encoding is context-dependent, and that objective representations of context are not possible.

Moving from a windowless office to a sunny outdoor terrace, for example, epitomizes the visual system’s adaptiveness to context. Sitting in an office, we see a colleague wearing a blue jeans and a green shirt. The light reflected from the blue jeans gives rise to perceptual experience based on approximately 10^17^ photons/s with a mean wavelength of 450 nm streaming off every square centimeter. In a similar way, about 10^17^ 550-nm photons/s/cm^2^ are streaming off of the green shirt. Next, we step outside into the bright sun with that colleague for lunch. On the outdoor terrace, he looks the same: blue jeans and a green shirt. However, in bright sun, this identical perceptual experience is being produced by around 10^23^ 450-nm photons/s/cm^2^ streaming off of the blue jeans and approximately 10^23^ 550-nm photons/s/cm^2^ streaming off of the green shirt. As Paul Glimcher (2011, p. 275) describes, “on a typical day this is a *six-order-of-magnitude* shift in the objective reality, which has been accompanied by no significant change in the subjective experience.” In other words, despite such a significant change in objective reality, we experience our observations the same. The reason for this stable experience should be obvious from an evolutionary perspective. The most important things we need to understand in order to survive are the objects and people in our immediate surroundings, not the sun located 150 million kilometers away. To extract the properties of our immediate context accurately, our senses have to subtract the changing effects of the sun as we move under clouds, into shadow, or into direct sunlight. This adaptation economizes the brain’s energy consumption and helps us to navigate effectively in different environments.

As another example, the Adelson’s Checker-Shadow Illusion presents two simultaneously perceived targets as identical local stimuli – that is, they are reflecting identical numbers of photons to the human eye. However, one is perceived as being lighter than the other (Glimcher, 2011). Again, a human will perceive the tiles as being different due to a spatial reference dependence triggered in the visual system by the differently shaded tiles that surround them (Glimcher, 2011). Similarly, in the table Illusion of Shepard (1990), people perceive one table to be longer and thinner than the other, even though they are the same size on the page.

In addition, other sensory encodings are reference-dependent, and nowhere in the nervous system are there objective representations of surrounding context. Because people receive much more sensory information than they can physically process, the sensory system’s central purpose is to help people navigate the 3D world. Then a sensory system can only make a “best guess” about the configuration of objects in the 3D world (Helmholtz, 1873; McKenzie et al., 2018). A sensory system draws on contextual cues within this system to construct this guess. Whereas behavioral economics (Thaler and Sunstein, 2009) use sensory illusions as examples of people’s shortcomings of our cognitive system, McKenzie et al. (2018) argue that illusions showcase the sophistication and adaptiveness of the human mind. Sensory processes help us to understand how the system gets things right, not wrong, in the real (3D) world.

If a consumer does not follow the rules of traditional economic models, what are the rules behind consumer behavior? According to a contextual approach, optical illusions and other behavioral biases are manifestations of the human ability to adapt *effectively* to the environment. These adaptive skills need not be exact copies of the world; approximations are enough. There is good reason from an evolutionary psychological perspective, for a human’s mental processes to optimize an inflow of information by making approximations, rather than objective copies of the environment. Unexpectedly, many patients with neurophysiological deficits perform better in perceptual illusion tasks than healthy participants. For example, Fine et al. (2003) describe a sight-recovery patient (“MM”) who perceived Shepard’s Tables to be the same size. MM was blinded at age 3 and like other sight-recovery patients, had difficulty with 3D interpretation of retinal images. Similar deficits in approximation during different visual perception tasks have been observed in schizophrenia patients (Dakin et al., 2005), patients with memory deficits (Edin et al., 2009), and among autistics (Heeger, 2017). The fact that different kinds of patients do not fall prey to visual illusions is indicative of an impaired visual or other mental system, rather than an ideal one (Heeger, 2017; McKenzie et al., 2018).

Consistent with arguments made by McKenzie et al. (2018), this article argues that interpretations of consumer’s decision-making as being irrational are sometimes misguided in ways that are analogous to the interpretation of visual and other sensory illusions as being indicative of a shortcoming of the visual and other sensory systems. What appear to be violations of original choice theory turn out to reflect adaptive responses to relevant information in a decision-making context.

Traditional models of consumer choice assume that an ideal consumer chooses based on his or her explicit, fixed beliefs and preferences. However, it is well-known that although human representations are routinely at odds with physical measurements of real world properties (Purves et al., 2015), these representations lead to effective behaviors and decisions (McKenzie et al., 2018). According to a contextual approach, the task of human representation is not to recover properties of the world in a traditional, logical sense, but rather to cope with different situations by promoting useful behaviors in life. Then consumers’ representation of information reflects biological, social, and cultural experiences as well as the most important patterns of the current environment rather than objective features of the environment in an objective sense (Barkow et al., 1992; Purves et al., 2015; McKenzie et al., 2018; Martens, 2019).

### The Consumer’s Contextual Decision-Making Model

Despite the fact that traditional economic models have excluded context when explaining consumer behavior, there are many psychological and neuroeconomics studies that have shown that consumer behavior is strongly context-dependent (Louie and De Martino, 2014). Axiomatic contextual approaches have also been developed within traditional economics that view consumers’ willingness to pay for a good not as fixed, but rather dependent on the market environment and how they respond to it (Kőszegi, 2010). The meaning of “context” in this sense varies from a single stimulus and option sets to a social structure. Consumers are sensitive to the size of choice sets, the order of options, and the relationships between the different options within a choice set (Louie and De Martino, 2014).

In addition, psychologists have recognized that the representation of social choice sets affects which target features become most salient, and as a consequence, how each constituent individual or social group within the choice set is evaluated (Chang et al., 2019). Furthermore, mutual understanding is often structurally built into context. For example, trust is based on the shared belief that each person will behave accordingly in a specific context. Bacharach and Gambetta (2001) have termed the degree of trust in a given context as its “trust-warranting properties.” In most situations, trust is formed based on considerations other than pay-offs. Many social properties, for example, increase trust, such as commonly-held values, perceived honesty, benevolence, and cultural dispositions and practices (Keren, 2007). Thus, the concept of context includes physical and social functions, structures, and their interactions. According to the CCDMM, a consumer learns to predict these context-specific properties.

The CCDMM assumes that consumers do not act without considering the likely consequences of their actions. They do not waste effort planning for future contingencies that are very unlikely to happen (Jaynes, 2003). Consumers anticipate properties in different contexts based on prior beliefs and experiences. The consumer uses these previous experiences, which we call “priors” in the Bayesian language, to processes information in a specific context. Figure 1 presents the CCDMM.

The previous contextual approaches typically assume that consumers’ decision-making begins based on stimuli – in this case any goods on the market, as shown in Figure 1 (Gershman and Niv, 2013; Tymula and Plassmann, 2016; DuBrow et al., 2017). On the contrary, it is essential, according to the CCDMM, that the mental context (e.g., Cm_2_ in Figure 1) is the starting point for the consumer’s decision-making. Decision-making is based on either SIMS or WOWS.

A marketing context is formed of elements that have an internal structure. Elements that belong to the same context have a high frequency of spatial and temporal co-occurrence compared to the elements that do not belong to the same context (Schapiro et al., 2016; Tymula and Glimcher, 2016). Our contextual mental models (Cm1, Cm2…Cmn in Figure 1) organize and structure continuous experience. These context representations allow a consumer to apply his/her marketing knowledge (i.e., PB’s in Figure 1) across time and space (Franklin et al., 2020).

The growing literature suggests that the hippocampus is especially important for the learning and extraction of internal structures in the environment (Schapiro et al., 2016). When an individual has a Cm_2_ and begins to make decisions concerning a birthday party, he/she forms expectations about common elements concerning the birthday party. SIMS means that an individual’s decision-making is based on a correspondence link between expectations and the real market context, and the value of this correspondence link is high. It is noteworthy, that the internal structure of a context does not only include only physical properties, but also their functions and symbolic meaning relate to this specific context. It is still an open question of how the brain forms this correspondence link between PB’s and the market context. However, there is consensus that the traditional model based on a one-dimensional scalar function is not enough in order to describe and explain this complex process. Dayan (1993) introduces the concept of “the successor representation,” which is a combination of the initial state and the destination state. From the perspective of CCDMM, the initial state is CM, the destination state is the marketing context and the correspondence link is the successor representation. Thus, a promising direction, in the vein of Dayan’s model, will be to assume that both a PB and the real market context can be described by vector spaces and then the correspondence link between these two vector spaces can be counted computationally and experimentally in order to test CCDMM in a market-like context.

In line with the above argument, the WOWS means that an individual’s decision-making cannot rely on the correspondence link between expectations and the real market context, because these two do not correspond strongly enough. What is a sufficient correspondence in order to apply SIMS or WOWS? That is a difficult question, because all information is novel to some degree and we never encounter anything twice in a strict sense (Bar, 2011). If we think that number 1 describes the total similarity between PB and the real market context and number 0 describes the complete difference between these, then there should be a specific equilibrium between PB and the market context for SIMS between 0 and 1. It is likely that this equilibrium – correspondence mapping between PB and the real market context – is closer to 1 in the case of SIMS. The argument here is that human behavior is driven according to the free energy principle, which means that our brains favor predictions, which are most probable in the market context (Seth, 2015). Therefore, the theoretical hypothesis here is that the equilibrium state should be biased to 1. When this equilibrium state begins to become unstable, an individual needs to apply WOWS and update the Cm relating to this context.

Although it is too early to determine what the exact equilibrium point (i.e., the value of the correspondence link) between the vector space of PB and the real market context is, when an individual needs to update Cm, and how much he/she needs to update it (a little evolutional change or a big revolutional change), there is some promising direction in the current studies relating to reinforcement learning (Takahashi et al., 2017; Franklin et al., 2020). These studies have shown that dopaminergic neurons in the brain are not only sensitive to value shift – when the context-related reward is better than expected – but also to the identity shift. These identity shifts are driven not only by the reward, but also by the content features of the context. Thus, there is neural correspondence between individual expectations and features of the context elements; this could be one neurophysiological trigger that upsets the equilibrium. In addition, the functional connectivity increases between the mPFC and hippocampus when participants in the experiment are in the transition phase between one context and the other (Schapiro et al., 2016). These findings of neurophysiological mechanisms when an individual changes his/her behavior relating to the changes in the context open new possibilities to determine the transition mechanisms of consumers, for instance, when they change their strategies from SIMS to WOWS. However, more behavioral and neurophysiological research is needed in order to prove this idea in a consumer decision-making context.

In a real market context, consumers use SIMS, because it is rational to assume that suitable commodities are available in a real market. In a real life problem requiring decision-making, most consumers have a reasonable idea of their prior beliefs, and because these beliefs are based on all of their past experiences, these prior beliefs experiences are not easily changed and are fairly stable (Jaynes, 2003). Most of a consumer’s decision-making takes place in these kinds of regular and repeated contexts. However, when prior knowledge of context is minimal or limited, the best a consumer can do is construct an interpretation of the properties of the context and make a decision based on contextual information. In other words, the consumer applies WOWS in a new context. Whereas SIMS works in most real market contexts, most decision-making studies have been executed according to WOWS, in which participants cannot apply their prior beliefs.

## SIMS, WOWS, and Contextual Rationality

In real-life contexts, offers on the market include more information than the consumer can access, and the brain needs to apply an effective strategy to concentrate only on meaningful information. Whereas the concept of meaningfulness refers to the degree of significance an individual generally assigns to information, meaningfulness is still rooted in culture and in the values and attitudes it conveys. Then, the concept of meaningfulness has two components, the subjective goals and recognition (Ariely et al., 2008). The subjective goal refers to mental representations of potential future situations. These representations enable a person to produce control-related decision-making strategies (Geary, 2005). For example, when young people pursue the goal of graduating from university, they form subjective goals about their future earnings and these expectations have an important impact on their related decisions (Suomala et al., 2017). Recognition means that an individual’s decision-making and behavior are socially and culturally acceptable and valued. Thus, the meaningfulness has two components: personal goals and recognition. According to this approach, the rational decision maker does not recover objective properties of the world, but copes with different situations by promoting meaningful behaviors in life. The decision maker’s representation of information reflects the meaningfulness of the situation based on past experiences rather than objective features of the environment (Barkow et al., 1992; Geary, 2005; Purves et al., 2015; Martens, 2019).

Therefore, human behavior is biased to socially and culturally transmit values and attitudes. Thus, most consumers anticipate something “meaningful” to happen in specific contexts, and this leads to the SIMS.

However, when the typical anticipation for a specific context is disrupted, a consumer tries to understand the context based on available information. Thus, a consumer constructs a new model for the context by updating his or her prior beliefs based on contextual information and tries to find balance between uncertainty and prior beliefs using WOWS.

Three typical cognitive biases will be presented and interpreted based on the CCDMM. First, the example of *anchoring* illuminates how a consumer tries to balance prior beliefs and new contextual information. The anchoring effect is a cognitive bias that describes the common human tendency to rely too heavily on the first piece of contextual information offered (the “anchor”) when making decisions (Tversky and Kahneman, 1974). Once an anchor is set, an individual will make subsequent decisions by interpreting information around the anchor. For example, the initial price offered for a house sets the standard for the rest of the negotiations, so that prices lower than the initial price seem more reasonable even if they are still higher than what the house is really worth.

In the classical experiments by Tversky and Kahneman (1974), participants were given an arbitrary number between 0 and 100 and asked to indicate whether the percentage of African nations in the United Nations was higher or lower than that number. Research participants then estimated the actual percentage. Results indicated that participants who had received a relatively high number as an anchor for comparative judgment gave higher absolute estimates than participants who were given a lower number as an anchor of comparison. If we accept that the anchor somehow infiltrated participants’ prior beliefs, then it would have pulled estimates up or down because they did not possess any prior beliefs on the topic in this experiment. Therefore, it is a very relevant anchor. Thus, they applied WOWS in their attempts to understand contextual meaning. As another example, often rely on experts when it comes to a topic like scientific information being presented in media contexts, because they lack the relevant background information to make their own decisions (Bromme and Goldman, 2014). Thus, WOWS is a tool to cope with new and unfamiliar situations.

Second, *framing effects* occur when people’s decision-making systematically depends on which logically equivalent description of outcomes or objects is presented to them. Framing effects violate the principle of a traditional model of economics, according to which logically equivalent descriptions should lead to identical decisions (Levin et al., 1998). For example, ground beef can be described as containing 75% lean meat or, alternatively, 25% fat meat (Levin and Gaeth, 1988). Although the two terms are logically equivalent (describing exactly the same sort of meat), they are not, what McKenzie and Nelson (2003) refer to as “informationally equivalent.” A butcher who advertises his meat as 75% lean delivers his customers a slightly different message than his counterpart who advertises his meat as 25% fat. Specifically, by stating that the ground beef is 75% lean, the butcher may be highlighting a positive feature, the one that the customer presumably wants to maximize. The butcher is thus signaling that he is aware of the customer’s desire and attempting to satisfy this need. The other butcher, who advertises his meat as 25% fat, is emphasizing a negative feature, indirectly signaling lack of concern for the customer’s desires.

Finally, the *decoy effect* (also called the *attraction effect* or the *asymmetric dominance effect*) is a phenomenon in which consumers will tend to have a specific change in preference between two options – target and competitor – when also presented with a third option (the “decoy”) that is inferior in all respects to both the target and the competitor (Huber et al., 1982). A decoy option is asymmetrically dominated because it is inferior in all respects to one option (e.g., the target), but only inferior in some respects and superior to another option (e.g., the competitor). The decoy effect violates the independence of irrelevant alternatives (IIA), in particular, an axiom which holds that if an individual prefers, for example, pizza to a hamburger, when considering the choice set (pizza and hamburger), then he or she also prefers pizza to hamburger in any other choice set (e.g., pizza, hamburger, and spaghetti). In other words, the relative probability of choosing pizza to the probability of choosing hamburger should be the same according to the IIA, independent of whether spaghetti is available or not (Luce, 1959; Ray, 1973). Thus, the decoy effect is one of the contextual factors which affect consumer decision-making and has been exploited by marketing and political strategies (Lehmann and Pan, 1994; Dhar and Glazer, 1996) as well as in other contexts (Louie and De Martino, 2014). Louie and De Martino (2014) have further hypothesized that the decoy effect is the influence of a common biological mechanism, because similar effects have also been observed among multiple animal species.

In most of the experiments where cognitive biases have been demonstrated, participants are in situations where they cannot apply SIMS. This means that they need to construct a contextual model based on signals from the context, not from previously acquired beliefs. One possible explanation for this is that participants reveal their first impressions of a problem requiring decision-making in these cognitive bias studies, whereas decision-making in a real-world context is not grounds for first impression reactions. However, if the problem in the experiment allows use of prior knowledge and experience, cognitive bias effects decrease or disappear (List, 2003; Leong et al., 2017). In addition, in many cases, experimental control is often gained at the expense of ecological validity; participants cannot apply SIMS or change from SIMS to WOWS (and vice versa). These traditional experiments leave the question of what prior beliefs consumers use in a real market environment unanswered.

It is important to emphasize that WOWS is also rational from the CCDMM perspective. When a human has the tendency to look for relevance in a new or radically changed context, he or she tries to find a reference point in the uninterrupted commotion and interpret the situation in a meaningful way. Then, if a task includes the wording “the ground beef is 75% lean,” an individual tries to interpret this wording from the point of view of either the experimenter and/or the butcher (Leong et al., 2017). In this way, context can “leak” information about the experimenter’s intentions, and these signals are different in different options, despite options being logically equivalent. In short, contexts carry information beyond their literal content (McKenzie and Nelson, 2003).

Moreover, when considering the example of the daughter’s birthday in Figure 1, it is intuitively difficult to conceive of how the anchor and other cognitive biases would affect an individual’s decision-making in this context, because the contextual mental model and prior beliefs are related to “daughter’s birthday party,” a well-known cultural and social event. In addition, consumers usually possess existing prior knowledge about the commodities available in their living environment. Moreover, in situations like these, most people make decisions using SIMS. A general problem with traditional and behavioral economic models is that they concentrate on situations in which people need to apply WOWS, whereas most of the time, people in a real market context apply SIMS. It is plausible on a hypothetical level that traditional reinforcement learning procedures do not explain decision-making based on SIMS. Thus, there is a lot of space for new avenues of study in these directions.

The essential question is, how does a consumer rationally constrain his/her decision-making process in an information rich environment? The promising candidates for this “rational framing machine” in the brain are the reference point and the ecological rational approach. Kahneman and Tversky (1979) have conceptualized this as status quo, but recently growing evidence has suggested that the reference point is better described as an outcome expectation (Tymula and Glimcher, 2016; Suomala et al., 2017). The reference point is likely not a one-dimensional value scale, but a many-dimensional vector scale instead. Another direction of rational approach to cognitive biases comes from the ecological rational school founded by Gigerenzer (2008), who emphasizes that heuristics are rational most of the time, because a decision maker has limited time and cognitive resources. The reference point as both an outcome expectation and ecological rationality are consistent with the argument of this paper, that the context-specific expectations or prior beliefs constrain the interpretations of coming information of the specific context. More research is needed in order to describe and explain how consumers form reference points or use rational heuristics in a Gigerenzer sense in a real market context.

People parse their decisions based on what is similar and what is different. Current research emphasizes that humans cluster experiences together based on similarity in particular (Gershman and Niv, 2013). In a specific problem requiring decision-making, a consumer anticipates the structure of the problem’s context by clustering structure, functions, and interactions of this context. Thus, humans organize their knowledge into discrete units called *chunks* based on regularities and similarities (Gershman and Niv, 2013; Gershman et al., 2017). In other words, consumers attempt to cluster their experiences based on the similarity of their interactions with this environment in the past. In addition to the representation of the physical context, similarity also works in the social environment. For example, a salesman who reported his own paint consumption to be similar to a customer’s sold a larger quantity of paint (Brock, 1965). Furthermore, social influence is stronger when it originates in someone or something that is similar to the person being influences than from someone or something dissimilar (Gershman et al., 2017). The default assumption is that a consumer tries to start with SIMS. However, when consumers do not have any prior knowledge of a context or if the properties of a context change radically, it is necessary to create new categories, and they apply WOWS.

A rational consumer concentrates only on relevant marketing information. The brain’s information processing is metabolically very costly (Lennie, 2003); our brain tissue is about seven times as “expensive” as the average tissue in our bodies (Tymula and Glimcher, 2016). Given a fixed neural activity budget, an efficient neural representation of a context would aim to increase discriminability between the most relevant inputs (Woodford, 2012). Then the essential question is what qualifies as relevant information and what is the simplest possible amount of information needed to achieve a result. The answers depend on the properties of the context and on the consumer’s personal goals. For example, in an educational context, it is important to design multimedia so that it supports meaningful learning (Mayer, 2009). Then, the simplest possible amount of required information is determined by the learners’ visual and verbal cognitive capacity and the essential knowledge relating to the learning content. In this example, relevance is related to the most essential features of the educational context. The rational behavior is related to the scarcity of information, and the most important action in this process is to discard extraneous information and focus on the most relevant aspects of the context. The educational context is a solid illustration of an individual’s decision-making because the goals of learning have been determined in a curriculum formed by educational policy-makers and educational experts, not by individual learners. However, if a learner does not feel that the learning content is relevant, his or her learning process will be disturbed.

In addition, the notion of relevance has specific meaning in models for human decision-making. Current research highlights that decision-making becomes relevant when the reason for a choice is to fit important personal goals (Csikszentmihalyi, 2000), which can shift across life stages (Bhattacharjee and Mogilner, 2014). Furthermore, relevance is defined as the set of contrasts a consumer is able to make with respect to the distinctions between options in the market (Ratneshwar et al., 1987). Thus, meaningfulness and relevance are the results of a person’s capacity to interpret the properties of context in which they may make decisions. Ratneshwar et al. (1987) suggest that the relevance of stimulus objects in a given context is a function of the human capacity to concentrate on the most meaningful features of the environment in order to navigate it.

The anticipation of essential features of a market context helps a consumer to concentrate on its most relevant features. It is rational to begin with prior beliefs about the most regular and repeated properties of a context – that is, by applying SIMS. This assumption is consistent with the notion that the common goal of brain functions is to satisfy a “free-energy principle” (Friston, 2010) and approach with “efficient coding of subjective value” (Tymula and Plassmann, 2016; Polanía et al., 2019). When consumers encounter a new situation, they interpret it based on the information obtained from the situation, and apply WOWS.

## CCDMM and Neuroeconomics

Common knowledge holds that when purchasing a new house, a laptop, or a pair of shoes, people generally believe that conscious deliberation increases the likelihood that they will make the right choice. However, recent insights show that often the “deliberation-without-attention” leads to better decisions and satisfaction levels among consumers than does conscious deliberation (Dijksterhuis et al., 2006). Furthermore, current research has shown that behavioral science methods do not provide a sufficiently comprehensive picture of consumers’ actual decision-making. However, work in the field of neuroeconomics has found promising evidence for more accurate methods of obtaining more exact knowledge from consumer behavior. Section Problems of Previous Consumer Science Methods describes the main problems of behavioral science methods; section The Brain’s Valuation Network then describes how neuroeconomics provide more accurate ways of describing and explaining consumers’ behavior from the CCDMM perspective.

### Problems of Previous Consumer Science Methods

Self-reporting surveys and interviews are widely used tools for consumer research. Surveys of focus groups have been used to predict the success of new products, services, TV shows, ad campaigns, and even public health interventions (Scholz et al., 2017). These methods usually ask consumers about their intentions to change a behavior, to buy new commodities, their level of self-efficacy, or their beliefs about their own behavior (Venkatraman et al., 2015). Although the use of these self-reporting measures (questionnaires and interviews) are common, they are not perfect predictors of consumers’ behavior in a real market context. Often the predictive power of these traditional methods is weak, as demonstrated by the fact that between 75 and 95% of all new products fail (Schneider and Hall, 2011).

The *verbal overshadowing effect* refers to the impairment of object recognition in subsequent tasks as a result of verbal explanation. For example, studies have shown that people who verbalized the pros and cons of an object made worse decisions because doing so prevented them from gaining access to their “gut feelings” about alternatives (Wilson and Schooler, 1991; Wilson et al., 1993). Furthermore, people demonstrated considerable levels of choice blindness, even for remarkably different tastes like cinnamon-apple and bitter grapefruit jams. This occurs because people verbalized their choice before researchers secretly switched the content of the sample containers (Hall et al., 2010). In addition, it has been confirmed that consumers’ purchases of complex products were viewed more favorably when decisions had been made in the absence of verbalization and attentive deliberation (Dijksterhuis et al., 2006). Asking consumers how much they like something requires several mental and neurophysiological operations, including the initial processing of the stimulus, referencing similar items with which the consumer has experience, and projection of future benefit, all of which may be subject to the mental overshadowing effects of the experiment. Thus, while the act of rating something requires a verbal process, the brain response during the consumption of the good does not, and the latter may prove superior to rating approaches (Berns and Moore, 2012).

Therefore, it is not surprising that research methods based on linguistic processing do not yield a sufficiently precise understanding of consumer behavior. These traditional measures represent *post hoc* introspection about experiences from an earlier stimulus and could thus be distorted by a variety of factors, including higher cognitive processes and emotions (Venkatraman et al., 2015). Instead, current neuroeconomic research has shown that much of the normative attitudes and preferences expressed in life is processed subconsciously within the brain, and is not easily accessible for verbal self-report (Camerer et al., 2005; Venkatraman et al., 2015). This current approach has shown that decision-making involves, besides deliberative processes, many subconscious mental and neurophysiological processes like concepts, sensory encodings, personal issues, situation-specific information, valuation, and emotions (Schröder et al., 2014; Tymula and Glimcher, 2016; Genevsky et al., 2017). These neurophysiological processes that give rise to behaviors occur in different regions of the brain simultaneously and are not always accessible to awareness (Cooper et al., 2015). Therefore, people often have a limited ability to consciously identify why they do what they do. It is difficult to argue, therefore, what their real personal preferences are. Despite these problems, the mental processes underlying decision-making are nevertheless represented in the brain (Berkman and Falk, 2013).

Neuroeconomics uses mainly brain-imaging techniques, in particular functional Magnetic Resonance Imaging (fMRI) to bypass the weakness of self-reporting and observe the brain activity that underlies particular consumer behavior. The fMRI scanner generates a strong static magnetic field and can reveal changes in blood flow when a participant is lying inside a large chamber, allowing researchers to study neural activity in the human brain almost in real time (Ashby, 2011; Suomala, 2018b). Therefore, it is no wonder that fMRI has grown to become the dominant measurement technique in cognitive neuroscience and neuroeconomics (Ruff and Huettel, 2014). FMRI measures the blood oxygen level-dependent (BOLD) signal, which is a measure of the ratio of oxygenated to deoxygenated hemoglobin. By using fMRI, researchers can infer which brain areas consume more oxygen and sugar than do inactive areas. These methods allow scientists to infer what happens within the brain when people are exposed to a certain product, advertisements, or other stimuli in a specific context. It is important though, to connect the neurophysiological data to real behavior, because human behavior is the result of coevolution of neurophysiological, mental, and social issues. There are also other neuroscientific techniques in consumer neuroscience and neuroeconomics (Suomala 2018a,b), however, as most of the scientific studies relevant to the topic were executed by fMRI, it is on this method that the articles focus.

The main task of the human brain is to effectively serve the host’s biological, psychological, and cultural needs (Yamins and DiCarlo, 2016). Then the central insight of CCDMM is – in the same vein as evolutionary psychology and neurophysiology (Barkow et al., 1992; McDermott et al., 2008; Purves et al., 2015; Wilson et al., 2018) – that the essential task of the brain is not to copy facts but rather to help a person cope with everyday situations. This process can be best understood within the context of people living their everyday lives. Research in neuroeconomics has demonstrated that brain activity is a very dependable predictor of behavior. Using fMRI, scientists map the areas of the brain that respond to different types of stimuli in different contexts. These activity patterns in people’s brains have been shown to be good predictors of individual responses to ad campaigns and likelihood of conforming to social norms (Scholz et al., 2017). Recent studies have also shown that brain activity is not only a predictor of individual behavior, but also a better predictor of a representative population’s behavior than the traditional survey or focus group survey (Berkman and Falk, 2013; Genevsky et al., 2017). Measuring brain activity is therefore a new way to study consumers’ underlying beliefs and hidden information about their true preferences. As the field of neuroeconomics expands, the integration of brain imaging underlying brain function will complement the use of traditional behavioral surveys in helping us to better understand consumers’ decision-making in different contexts.

There are a number of reasons why neuroscientific methods and approaches should also be applied when developing better consumer theory, especially when examining the role of SIMS. In the following section, a neuroeconomic approach is presented from the CCDMM perspective.

### The Brain’s Valuation Network

Growing evidence from neuroeconomics shows that there are general decision networks in the brain, which count the total benefits (i.e., valuation) of different commodities in the market using a common neurophysiological currency. Whereas in many contexts, a number of variables (tone, color, characters, etc.) and attributes are involved in many market contexts and advertising messages, this complexity makes it almost impossible to isolate and measure the contribution of each variable using traditional methods. However, the brain’s valuation network completes this demanding task and forms a net value of commodities and other items in different contexts.

The brain activation changes in this valuation network correlate with a commodities’ values in a wide class of objects, from biological needs like food (Levy and Glimcher, 2012), clothing (Lim et al., 2013), and money (Glimcher, 2014) to abstract cultural values like charitable donations (Genevsky et al., 2013). The valuation network is made up of the Medial PreFrontal Cortex (MPFC), Ventral Striatum (VS), and Precuneus.

Studies have shown that small samples of participants’ brain activation profiles in neuroscientific experiments can predict real behavioral chance in a real context. A sunscreen study, for example, demonstrated that when subjects were exposed to persuasive messages concerning sun exposure, neural signals in the MPFC, could be used to predict changes in sunscreen use 1 week following the experiment (Falk et al., 2010). Moreover, neural signals in the MPFC predicted variability in behavior more accurately than self-report measures alone (Falk et al., 2010).

In another example, Falk et al. (2011) examined smokers’ neural responses to antismoking ad campaigns and subsequent smoking behavior. Consistent with the findings of the sunscreen study, when subjects were exposed to antismoke messages in the fMRI scanner, the MPFC activation in the brain more accurately predicted participants’ inclination to quit smoking 1 month after the initial fMRI than traditional behavioral measurements. Thus, activation of the critical valuation area in the brain (the MPFC) may serve as an indirect marker of future behavioral changes. In addition, activity in the same region as the MPFC that predicted behavioral changes during message exposure also predicted population level behavioral changes in response to health messages and provided information that was not conveyed by participants’ self-reports. Therefore, incorporating neural data with self-report measures may provide additional information for the development of predictive models. These results extend the use of neuroimaging to predict other types of behavior, as opposed to simply predicting immediate effects (Berkman and Falk, 2013).

In the same vein, by using fMRI it is possible to predict the success of new songs on the market. When subjects listened to unknown popular music in the scanner and evaluated these songs behaviorally, brain activation in the Striatum predicted the success of the songs in a real market context over the next 3 years, whereas subjective likability – measured behaviorally – was not predictive of sales (Berns and Moore, 2012). In addition, the fMRI-communication study identified neural regions associated with successful message propagation in the brain’s valuation network and in the brain’s metalizing system (the Temporal Parietal Junction, TPJ; Falk et al., 2012). In this study, initial idea recipients’ fMRI data profiles forecast an idea’s success beyond the initial recipients to others whose brains were never examined and whose eyes are never exposed to the original information.

The above described fMRI studies suggest that neural responses to messages and commodities within the valuation network of the brain are not only predictive of purchase decisions for those individuals actually scanned, but may also be generalized to the population at large and used to predict the success of the sales of products and effectiveness of messages. Activity in the valuation network of the brain clearly predicts the real world success of different advertising campaigns, products, services, and social messages at the population level, whereas self-reports, which have been the target of consumer research for a long time, are not as successful in their predictions.

Human behavior is the result of the coevolution of neurophysiological, biological, and social issues. Similarity-based reasoning has been studied neurophysiologically, when participants recognize, classify, or judge objects (Gershman and Niv, 2013; Wirebring et al., 2018). These studies have shown that the humans have a proclivity to cluster experiences together into contexts based on similarity, and the brain’s regions in the orbitofrontal cortex and precuneus play an essential role in similarity-based reasoning (Gershman and Niv, 2013; Wirebring et al., 2018). These findings are consistent with the basic idea of SIMS and the essential task of future research will be to compare whether consumers’ decision-making has similar neurophysiological mechanisms to similarity-based reasoning. Relating to the WOVS, it is difficult to find studies in which consumers change their strategy from SIMS to WOWS according to CCDMM.

As stated previously, the main task of the human brain is to serve the host’s biological, psychological, and cultural needs, and it serves not to copy facts but rather help a person cope with everyday situations. This is also the essential argument behind the CCDMM. The following section analyzes how the brain’s valuation network acts according to demands of different contexts.

As seen from the perspective of Occam’s Razor, consumer understanding is based on a very compressed representation of the world, and a rational consumer concentrates only on relevant marketing information. The brain’s valuation network might work according to this rule and rank the patterns of a context from the most important and indifferent to the least important things. What the valuation network needs to do is to consider many different attributes of each option (such as color, size, taste, and health benefits) and of its personal host (like how hungry or thirsty we are), assess the value of each of the attributes within a relevant consideration set, and most importantly, to combine all of these things into one coherent value representation that allows an individual to make decisions and behave in rational way. Current new axiomatic characterizations of how and why the brain tries to minimize metabolic cost is consistent with this idea about rationality (Steverson et al., 2019). Therefore, the brain must behave as though it represents the values of many different kinds of rewards on a common scale for comparison and choice. Regarding mental context, prior beliefs, and physical market context (Figure 1), a consumer tries to apply previously acquired knowledge and experiences as much as possible, or in other words, tries to save energy by applying SIMS. This principle is consistent with the free energy principle of the brain (Friston, 2010).

Furthermore, it is rational from the CCDMM perspective that a consumer represents the most essential patterns of contexts, and is flexible between different contexts. Context sensitivity is also typical for valuation network operations. When it guides valuation and decision-making, this neural process is modulated by several factors including the construction of the choice set, reward history, and perceived outcome relative to a reference point. A critical question for further research is whether contextual valuation coding might underlie context dependency at the behavioral level (Louie and De Martino, 2014). There are also studies about the neural mechanisms underlying decoy (Hu and Yu, 2014; Chung et al., 2017) and framing effects (De Martino et al., 2006).

The basic premise of the CCDMM is that a consumer makes predictions about the basic structure and functions of a context and their relationship to it when solving decision-making problems. Three building blocks behind CCDMM – inductive inference, the principle of Occam’s Razor, and Bayesian reasoning – work with predictions and help an individual to apply SIMS. Correspondingly, neuroeconomists measure brain activation in valuation networks while individuals evaluate information about various options, and then use that activation to predict subsequent behavioral outcomes, often over the course of weeks, months, or even years (Cascio et al., 2015; Knutson and Genevsky, 2018). Thus, the central tendency of the brain is probably to make predictions consistent with CCDMM.

## Discussion

This paper presents the CCDMM, which is a new interpretation of a consumer’s decision-making from a contextual perspective. Whereas traditional economic models do not provide framework for connecting effects to environmental properties and are silent about decision-making context, traditional behavioral models maintain a consumer’s context sensitivity as source of many cognitive biases. These models may seem contradictory and mutually exclusive, however CCDMM is not an alternative for traditional models; rather it may be viewed as an extension of them. However, when modeling a consumer’s decision-making with conceptual, computational, and mathematical tools, it is important to analyze this behavior in the real contexts. In some situations, a consumer faces one‐ or two-dimensional problem spaces, and it is suitable to apply “simple” economic utility functions (Jaynes, 2003). However, when one is faced – as is the case with most everyday problems – with an issue that includes many dimensions like emotions, attitudes, history of prior experience, and goals, then a fully adequate description of the human state and functions of mind would be better explained by studying brain functions. The brain has solved real-life problems during its evolutionary history and is able to adapt to a wide variety of contexts as a result. As such, it is possible to build a bridge between behavioral and neurophysiological approaches in order to better explain consumers’ decision-making.

According to the CCDMM, a consumer is actively making inferences based on prior experience and expectations not only about observable goods, but also about whole context (Baum, 2004; Gershman and Niv, 2013; Büchel et al., 2014). In order to build a more plausible model of consumer decision-making, this paper has presented shared principles in the mechanisms underlying subjective decisions and sensory perception (Louie and Glimcher, 2012; Woodford, 2012; Polanía et al., 2019). Models from this line of research have suggested that subjective value construction resembles sensory perception in that they are derived by inference processes that exploit information about the relevant properties of the environment (Polanía et al., 2019).

The CCDMM is consistent at a fundamental level with the models of logical thinking and rational reasoning of Jaynes (2003) and Baum (2004), as well as with evolutionary theory (Barkow et al., 1992; Geary, 2005; McDermott et al., 2008; Wilson et al., 2018). This paper shares current assumptions that people are cognitive misers, who aim to save time and effort when navigating the world (Tymula and Plassmann, 2016; Steverson et al., 2019). However, at the same time the brain is not passively waiting to see what will happen, but is actively making inferences based on prior experience and expectations.

In addition, The Proactive Brain model created by Bar (2007, 2009) has similar properties to the CCDMM. Bar’s model describes and explains the brain’s representation, classification and understanding of different objects in contexts without links to decision-making and choice in a traditional economics sense. The basic visual cognitive mechanisms of Bar’s model are the analogies, associations, and predictions, and it is the task of future research to determine how these mechanisms link to the decision-making mechanisms in the brain, when testing the CCSMM model behaviorally and neurophysiologically. Overall, the CCDMM provides suitable direction to further experimental, computational, and theoretical works.

The CCDMM has been described on conceptual levels. Whereas Bayesian reasoning is also a formal statistical tool used to study human decision-making, here it has been applied as conceptual framework. This conceptual framework gives directions for many testable hypotheses and experiments regarding consumers’ decision-making. First, it is important to formalize the basic concepts of the CCDMM. Then it will be possible to test the model’s operation computationally and experimentally. One interesting direction for research would be to examine what will occur when SIMS does not work in a specific situation, mental equilibrium gives way, and SIMS changes to WOWS. On a formal level, it is possible to count what the difference is between prior beliefs and the data in the context in which a consumer changes strategy from SIMS to WOWS. Graph theory (Markov et al., 2013; Suomala and Suomala, 2014) and the Bayesian network might be suitable tools for studying these ideas.

It has been argued that in most contexts, a consumer applies SIMS in an attempt to save mental and metabolic energy. However, on a general level, humans also have a tendency toward creativity and to produce new contexts. The limitation of the CCMDD is, therefore, that it does not describe a consumer’s behavior from a creative standpoint. Thus, another possible direction for future research would be to combine creative processes with the CCMDD.

## Data Availability Statement

The original contributions presented in the study are included in the article.

## Author Contributions

The author confirms being the sole contributor of this work and has approved it for publication.

### Conflict of Interest

The author declares that the research was conducted in the absence of any commercial or financial relationships that could be construed as a potential conflict of interest.
